# The role of acetylation in obesity-induced cardiac metabolic alterations

**DOI:** 10.3389/jpps.2024.13080

**Published:** 2024-07-23

**Authors:** Ezra B. Ketema, Gary D. Lopaschuk

**Affiliations:** Cardiovascular Research Centre, University of Alberta, Edmonton, AB, Canada

**Keywords:** obesity, cardiac energy metabolism, protein lysine acetylation, heart failure, fatty acid oxidation

## Abstract

Obesity is a growing public health problem, with its prevalence rate having tripled in the last five decades. It has been shown that obesity is associated with alterations in cardiac energy metabolism, which in turn plays a significant role in heart failure development. During obesity, the heart becomes highly dependent on fatty acid oxidation as its primary source of energy (ATP), while the contribution from glucose oxidation significantly decreases. This metabolic inflexibility is associated with reduced cardiac efficiency and contractile dysfunction. Although it is well recognized that alterations in cardiac energy metabolism during obesity are associated with the risk of heart failure development, the molecular mechanisms controlling these metabolic changes are not fully understood. Recently, posttranslational protein modifications of metabolic enzymes have been shown to play a crucial role in cardiac energy metabolic changes seen in obesity. Understanding these novel mechanisms is important in developing new therapeutic options to treat or prevent cardiac metabolic alteration and dysfunction in obese individuals. This review discusses posttranslational acetylation changes during obesity and their roles in mediating cardiac energy metabolic perturbations during obesity as well as its therapeutic potentials.

## Introduction

Obesity is a growing public health problem, with its worldwide prevalence rate having tripled in the last five decades [1]. Nearly 40% of the global adult population is overweight, while 13% of adults are clinically obese [1]. Similarly, the prevalence of both overweight and obesity has increased over fourfold in children [1, 2]. Overweight and obese accounts for over 4 million deaths each year worldwide [2, 3]. Obesity-related disability-adjusted life years (DALYs) has increased significantly in the last few decades, and the trend is projected to rise by 39.8% from 2020 to 2030 [3].

## Obesity and its burden on heart failure (HF)

Obesity is a major risk factor for various diseases, including cardiovascular diseases [1]. Cardiovascular diseases contribute to more than two-thirds of mortalities in obese individuals [2, 4]. Obese individuals also have a two times higher risk of heart failure (HF) development compared with subjects with a normal body weight [5]. It is projected that the prevalence of obesity will increase significantly in the coming years, while other risk factors for HF, such as hypertension, are expected to decline [1, 6, 7]. Regarding the specific HF pathologies, the incidence of HF with preserved ejection fraction (HFpEF) is far more common in obese individuals compared to the incidence of HF with reduced ejection fraction (HFrEF) [8]. Moreover, approximately 80% of HF patients with preserved ejection fraction are either overweight or obese [9].

Obesity leads to cardiac dysfunction and risk of HF development through both direct and indirect mechanisms. The indirect mechanisms include increased levels of circulating free fatty acids, pro-inflammatory cytokines, and adipokines that can lead to the development of metabolic risk factors which includes, insulin resistance, dyslipidemia, and diabetes [10, 11]. On the other hand, direct mechanisms of obesity-induced HF include myocardial lipotoxicity, changes in neurohormonal and hemodynamic balances, and microvascular dysfunction [10, 12]. Both direct and indirect mechanisms listed here are strongly associated with cardiac energy metabolic alterations seen in obesity. Alterations in cardiac energy metabolism, in turn, play a significant role in obesity-related HF development [13].

## Energy metabolism in the heart

**FIGURE 1:**
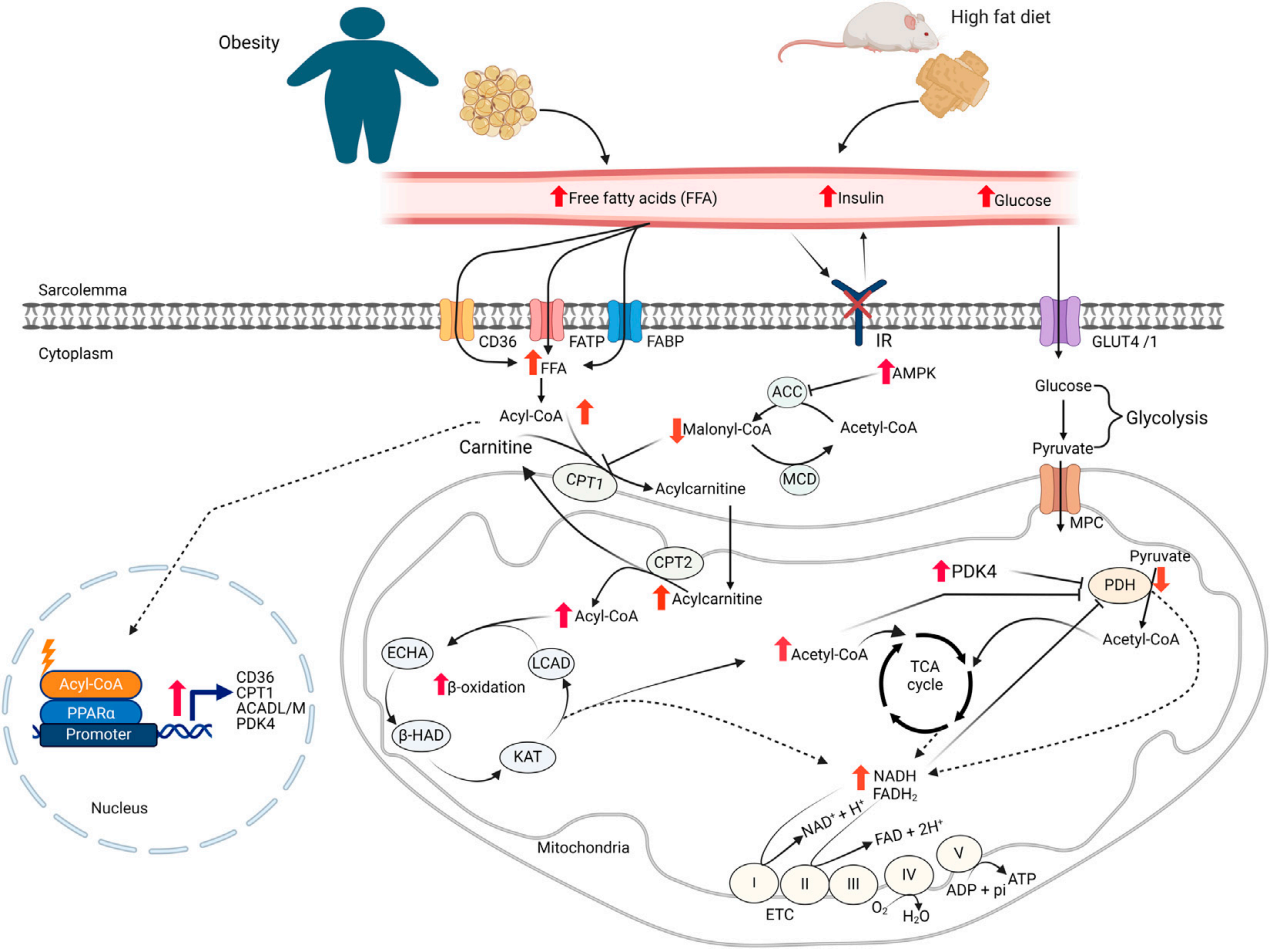
Cardiac energy metabolism changes in obesity FFA: free fatty acids; GLUT4, glucose transporter isoform 4; IR, insulin receptor; CD36, cluster of differentiation 36; FABP, fatty acid-binding protein; FATP, fatty acid transport protein; MCD, malonyl CoA decarboxylase; ACC, acetyl CoA carboxylase; MPC, mitochondrial pyruvate carrier; PDH, pyruvate dehydrogenase; LCAD, long-chain acyl CoA dehydrogenase; β–HAD, β-hydroxyacyl CoA dehydrogenase; KAT, 3-ketoacyl-coA thiolase; ECHA, enoyl-CoA hydratase; CPT, carnitine palmitoyltransferase; FAD/FADH_2_, flavin adenine dinucleotide; NAD/NADH_2_, nicotinamide adenine dinucleotide; ATP, adenosine triphosphate; ADP, adenosine diphosphate.

The heart has the highest energy demand of any organ in the body on a per gram weight basis [14]. In the healthy heart this high energy demand is fulfilled by metabolizing various fuel substrates, predominantly fatty acids and glucose, but also ketones, lactate, and amino acids depending on the supply, demand and neurohormonal states [15]. This ability of the heart to use different types of fuel for energy production is often described as “metabolic flexibility” or being “metabolically omnivorous.” Oxidation of fatty acids is the primary source of ATP production by the heart, accounting for approximately 60% of the ATP produced by the normal heart [14]. Myocardial fatty acid metabolism is regulated by both the supply of fatty acids to the heart, and by complex intracellular control mechanisms [14]. The intracellular control mechanisms involve allosteric, posttranslational, and transcriptional control of fatty acid oxidative enzymes [15]. The fatty acid supply and uptake into the myocardium is highly dependent on the circulating levels of fatty acids [16]. The subsequent uptake of fatty acids across the sarcolemma of cardiomyocytes is facilitated by at least three proteins: CD36, FA transport protein (FATP), and FA binding protein plasma membrane (FABPpm) (Figure 1) [17].

Within the cardiomyocytes, a key site involved in the regulation of fatty acid oxidation is at the point of mitochondrial fatty acid uptake by the enzyme carnitine palmitoyltransferase (CPT1) [14]. CPT1 facilitates the transport of long-chain fatty acids into the mitochondrial matrix by transferring the fatty acyl moiety from fatty acyl-CoA to carnitine to form acylcarnitines, which can be transported into the matrix for fatty acid β-oxidation. CPT1 activity is regulated by malonyl-CoA, a strong inhibitor of CPT1 [18]. Malonyl-CoA in the heart is synthesized from acetyl-CoA by acetyl-CoA carboxylase (ACC) and degraded by malonyl-CoA decarboxylase (MCD). Additionally, AMP-activated protein kinase (AMPK) regulates malonyl-CoA levels, and therefore fatty acid oxidation rates, by inhibiting ACC through phosphorylation (Figure 1) [19]. Once in the mitochondria, fatty acyl CoA undergoes β-oxidation, a repetitive and cyclic reaction sequentially catalyzed by long-chain acyl CoA dehydrogenase (LCAD), enoyl-CoA hydratase (ECHA), L-3-hydroxy acyl-CoA dehydrogenase (β-HAD), and 3-ketoacyl-CoA thiolase (3-KAT), until it is completely converted to acetyl CoA [14].

Cardiac glucose metabolism has two major steps. In the first step, glucose is taken up into cardiomyocytes through the insulin-dependent glucose transporter, GLUT4, or to a lesser extent, insulin -independent glucose transporter, GLUT1 [20]. In cardiomyocytes, glucose passes through glycolysis and converted to pyruvate. In the next step, pyruvate is transported into the mitochondria and converted to acetyl CoA by pyruvate dehydrogenase (PDH). In the end, acetyl CoA is oxidized to CO_2_ in the TCA cycle. In addition to fatty acids and glucose, the heart can also metabolize ketones and amino acids as sources of ATP [21, 22].

## Cardiac energy substrate metabolic alterations in obesity

Obesity occurs when an abnormal or excessive fatty acid accumulation occurs in adipose tissue due to imbalances in calorie intake and consumption [1]. It is associated with increased circulating levels of free fatty acids and triacylglycerols [23]. Increased levels of fatty acid transporter proteins on the sarcolemma of cardiomyocytes also occur in response to obesity [23–25]. Since myocardial fatty acid uptake is mainly influenced by the free fatty acid levels in the circulation [26], the increased levels of circulating fatty acids and fatty acid transporters on cardiomyocytes leads to an increased myocardial fatty acid uptake during obesity (Figure 1) [23, 25, 26]. Several pre-clinical and clinical studies have also shown that the increased myocardial fatty acid supply and uptake in obesity is accompanied by increased fatty acid oxidation rates in the heart (Table 1) [27, 28, 33, 34, 40, 41, 43, 44]. In addition to the increased fatty acid oxidation rates, excess fatty acid supply and uptake can lead to the accumulation of toxic lipid intermediates that have roles in the development of cardiac insulin resistance [45, 46]. The major lipid metabolite storage in the heart during obesity includes long-chain acyl CoAs, diacylglycerols (DAG), triacylglycerols (TAG), ceramides, and acylcarnitines [47]. The excess intra-myocardial storage of these lipid metabolites is associated with cardiomyocyte apoptosis, mitochondrial dysfunction, and lipotoxicity [13, 48].

**TABLE 1:** Alterations in myocardial energy metabolism in obesity.

Experimental models/conditions	Changes in myocardial energy substrate metabolism		
	Fatty acid oxidation	Glucose oxidation	Other findings
HFD-induced obesity in rats	Increased [14] Unchanged [24]	Decreased [14]	• Increased PDK4 [14] • Increased UCP3 expression [14] • Increased FA uptake [24] • Decreased glucose uptake [24]
Obese Zucker rats	Increased [25, 27]	-	• Increased FA uptake and esterification [25] • Decreased glycolysis rate [27]
*Ob/ob* mice	Increased [28–30]	Decreased [28]	• Increased FA oxidation gene [30] • Decreased glycolysis rate [29] • Increased AMPK phosphorylation [29]
*Db/db* mice	Increased [31, 32]	Decreased [31]	• Decreased glycolysis rate [31] • Increased in UCP activity mice [32] • Increased FA oxidation genes [32]
HFD-induced obesity in mice	Increased [33–38]	Decreased [35–38] Unchanged [39]	• Decreased glucose uptake [39] Decreased glycolysis [36] • Increased PDK4 and UCPs expression [36]
Obese patients	Increased [40, 41]	Decreased [42]	• Increased fatty acid uptake [40] • Decreased glucose uptake [41]

HFD: high fat diet; PDK4: pyruvate dehydrogenase kinase 4; UCP: uncoupling protein; FA: fatty acid.

The increased myocardial fatty acid supply and utilization in obesity suppresses cardiac glucose metabolism, a phenomenon known as the “Randle Cycle” [49]. In particular, myocardial glucose oxidation is markedly suppressed in obesity [28, 31, 35–37, 40, 42]. In addition to fatty acid oxidation-mediated inhibition of glucose oxidation, there are also several other mechanisms that can contribute to reduced glucose oxidation in the heart of obese subjects (Table 1). Firstly, the accumulation of lipid intermediates following excess supply or utilization is associated with the development of cardiac insulin resistance through different mechanisms [13, 29]. Secondly, the excess acyl CoAs in the heart of obese subjects is associated with decreased myocardial glucose uptake [29, 39, 41, 50]. Furthermore, increased acetyl CoA and nicotinamide adenine dinucleotide (NADH) production following high rates of fatty acid oxidation during obesity activates pyruvate dehydrogenase kinase 4 (PDK4), which inhibits PDH, the main enzyme of glucose oxidation, by phosphorylation [14, 36, 51]. As will be discussed, increased posttranslational acetylation of PDH may also be involved in this decrease in glucose oxidation.

Obesity can also affect myocardial fatty acid metabolism through transcriptional mechanisms. One of the important transcription factors controlling gene expression related to fatty acid metabolism is the peroxisome proliferator-activated receptors (PPARs). PPARs exist in three isoforms: PPARα (main isoform in the heart), PPARγ (predominantly in adipose tissue), and PPARδ. Increased levels of long-chain fatty acids in the myocardium are among the activators of PPARα. Activation PPARα has been shown to promote gene expression in myocardial fatty acid uptake, storage and oxidation [30, 52–54]. PPARα has also been shown to play a critical role in shifting energy substrate metabolism in the heart towards increased fatty acid utilization [55]. PPARα activation also suppresses glucose oxidation by increasing the expression of PDK4 [55, 56].

As discussed, obesity results in the heart becoming highly dependent on fatty acid oxidation as its ATP source, while the contribution from glucose oxidation significantly decreases [13]. This metabolic inflexibility is associated with reduced cardiac efficiency and contractile dysfunction [28, 57]. These adverse effects may be due, in part, to the fact that fatty acids are a less efficient energy substrate than glucose, leading to increased myocardial oxygen consumption per cardiac work [51]. The reduced cardiac efficiency due to high fatty acid oxidation rates is also associated with increased activity of uncoupling proteins (UCPs), which uncouples mitochondrial proton gradient from ATP synthesis by facilitating proton leak back into the mitochondrial matrix without generating ATP [32, 58]. Interestingly, increased activity and expression of cardiac UCPs have been shown in obese animals [14, 57–59]. An increased mitochondrial reactive oxygen species (ROS) production during lipid overload in obesity has also been shown to contribute to the increased activity of UCPs [32].

Although alterations in cardiac energy metabolism during obesity are associated with the risk of HF development, the molecular mechanisms controlling these metabolic changes are not fully understood. Tremendous efforts have been made to characterize the allosteric and transcriptional mechanisms contributing to altered cardiac energy metabolism in obesity. However, transcriptome and metabolomics studies revealed these mechanisms alone are not sufficient to explain the significant alterations in cardiac energy metabolism during obesity or heart failure [60, 61]. Recently, several posttranslational protein modifications have been shown to play a crucial role in cardiac energy metabolic changes seen in obesity. There are numerous reversible posttranslational modifications of proteins, including phosphorylation, methylation, acetylation, O-GlcNAcylation, ubiquitylation, succinylation, nitrosylation, SUMOylation, glycation, and β-hydroxybutyrylation. This review focuses on post-translation acetylation changes and their roles in mediating the cardiac energy metabolic perturbations during obesity.

## Posttranslational protein acetylation

Protein lysine acetylation is a reversible posttranslational modification that occurs by the addition of an acetyl group to the lysine residues of proteins. This acetylation modification alters the charge status on lysine residues, and adds an extra structural moiety, an acetyl group [62, 63]. This structural change impact proteins’ native structure, interactions with other proteins, stability, and function [64].

**FIGURE 2:**
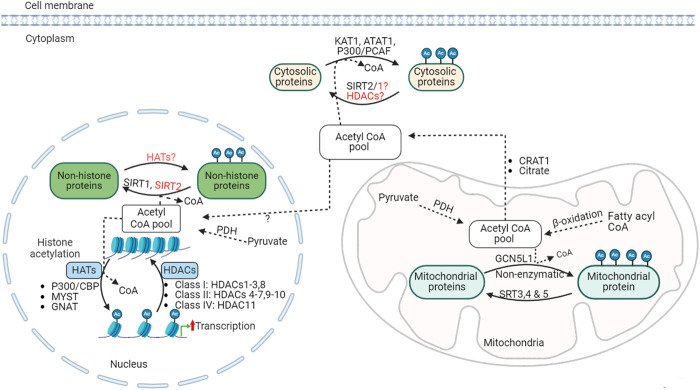
Acetylation regulation, acetyltransferases and deacetylases KAT1, lysine acetyltransferase; ATAT1, alpha-tubulin N-acetyltransferase; CBP, CREB-binding protein; PCAF, P300/CBP-associated factor; SIRT, sirtuin; GNAT, general control non-repressed N-acetyltransferases; MYST, MYST family acetyltransferase; GCN5L1, general control of amino acid synthesis 5 (GCN5) like-1; HAT, histone acetyltransferases; HDACs, histone deacetylases; PDH, pyruvate dehydrogenase; Ac, acetylated.

Posttranslational protein acetylation was identified initially on histone proteins over half a century ago [65]. Since then, histone acetylation modification has been widely recognized as an important epigenetic mechanism that regulates the structure of chromatin and gene expression processes (Figure 2). Dysregulation of histone acetylation is linked to altered gene expression profiles and has been implicated in several diseases, including cancer and metabolic diseases [66]. More recently, non-histone protein acetylation was also identified as an important entity in regulating cellular function [67]. With the help of advances in mass spectrometry-based acetyl proteomics, research in non-histone protein acetylation has expanded remarkably, leading to the discovery of thousands of lysine acetylation modifications in the cytosolic and mitochondrial proteins. Interestingly, many of these acetylated proteins are involved in energy substrate metabolism, including fatty acid and glucose oxidation [68, 69], (see [70] for review). However, even though it becomes apparent that non-histone protein acetylation is abundant, the exact contribution of these acetylation modifications to metabolic enzyme activity and metabolic flux regulation remains incompletely understood. In this review, we discuss recent progress made in understanding the role of posttranslational protein acetylation modification in relation to obesity-induced cardiac metabolic alterations.

## Obesity and changes in protein acetylation

**FIGURE 3:**
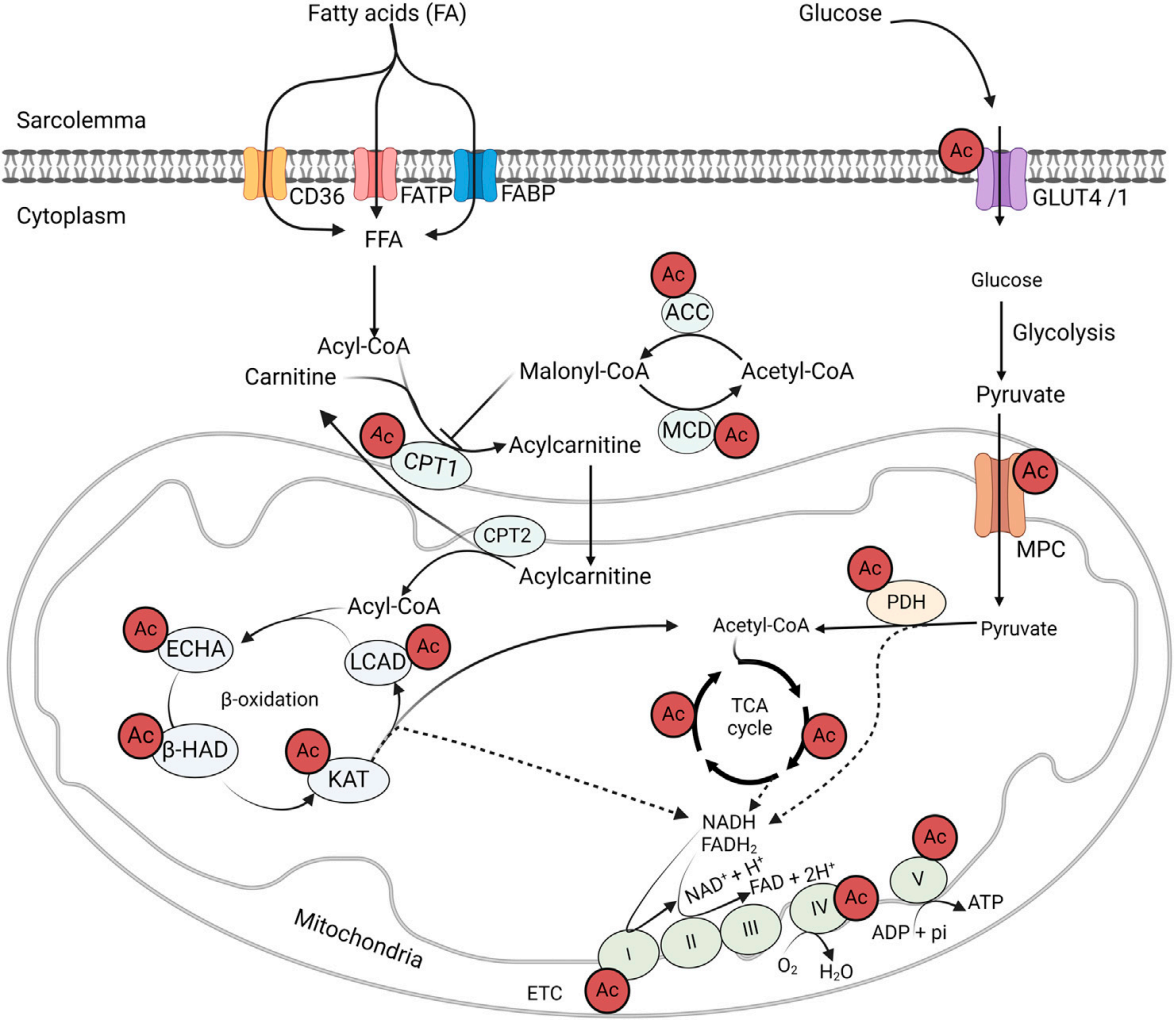
Acetylation control of cardiac metabolic enzymes and proteins GLUT4, glucose transporter isoform 4; CD36, cluster of differentiation 36; FABP, fatty acid-binding protein; FATP, fatty acid transport protein; MCD, malonyl CoA decarboxylase; ACC, acetyl CoA carboxylase; MPC, mitochondrial pyruvate carrier; PDH, pyruvate dehydrogenase; LCAD, long-chain acyl CoA dehydrogenase; β–HAD, β-hydroxyacyl CoA dehydrogenase; KAT, 3-ketoacyl-CoA thiolase; ECHA, enoyl-CoA hydratase; CPT, carnitine palmitoyltransferase; FAD/FADH_2_, flavin adenine dinucleotide; NAD/NADH2, nicotinamide adenine dinucleotide; ATP, adenosine triphosphate; ADP, adenosine diphosphate.

Numerous studies have demonstrated that obesity induces significant alterations in protein acetylation patterns, and suggesting that these changes may play an important role in the pathogenesis of obesity and obesity-related metabolic dysfunction (Figure 3) [71]. Accordingly, we demonstrated hyperacetylation of a number of myocardial metabolic proteins in obese mice induced by high-fat diet (HFD) feeding [35]. Similarly, hyperacetylation of mitochondrial protein has also been shown in obese subjects with HF in murine obesity models and human patient samples [72]. Dysregulation of acetylation proteins is also positively correlated with BMI values and mitochondrial dysfunction in obese-induced HF patients [72]. In another study, a large number of cardiac hyperacetylated proteins due to obesity were shown in a Zucker diabetic fatty/spontaneously hypertensive heart failure F1 (ZSF1) rat model of HFpEF [73]. The majority of these hyperacetylated proteins were related to fatty acid metabolism and other energy-generating pathways [73]. Similarly, several other studies have also shown that a HFD in mice leads to the hyperacetylation of several liver proteins involved in glucose and fatty acid metabolism [74, 75]. Pathway analysis of the hyperacetylated proteins in response to obesity also revealed the association of these acetylated proteins with metabolic dysfunction and cardiac remodeling [76].

Significantly increased histone acetylation levels have been shown in obese individuals with insulin resistance compared to lean individuals [77]. An association between HFD feeding and altered histone acetylation patterns has also been demonstrated in the liver [71, 78]. Interestingly, histone acetylation changes following HFD result in differential expression of genes associated with metabolic syndrome and NAFLD [71], highlighting the impact of histone acetylation changes in response to HFD on metabolic dysregulation. Some studies reported a dose-dependent increase in histone acetylation levels in response to acetyl CoA supplementation [79]. However, others reported different effects of acetyl CoA levels on histone acetylation across various tissues. For instance, acetyl CoA levels were correlated with histone acetylation changes in white adipose tissue and pancreas but not in the liver [80], indicating tissue-specific variations in histone acetylation patterns in response to dietary changes. In contrast, other studies in western-diet (WD) fed mice demonstrated decreased acetylation of hepatic histone proteins [81]. However, a recent study showed an enhanced histone hyperacetylation in the liver in response to chronic high carbohydrate HFD feeding, suggesting a different impact on dietary sources [82].

## Links between acetyl CoA levels and protein acetylation during obesity

Acetyl CoA is a common intermediate in fuel metabolism pathways, and is also an acetyl group donor for acetylation modification. Acetyl CoA is produced in the mitochondria from catabolism of fatty acids, glucose, lactate, ketones and amino acids. As discussed earlier, HFD and obesity are associated with an increased rates of mitochondrial fatty acid ß-oxidation, leading to excess acetyl CoA generation [13]. This excess acetyl CoA has the potential to drive hyperacetylation of mitochondrial proteins. Using radioisotope tracing experiments, previous studies have demonstrated that acetyl CoA generated from fatty acid ß-oxidation is a key driver of mitochondrial hyperacetylation [83], indicating the association between fatty acid ß-oxidation and protein hyperacetylation. It has also been suggested that high acetyl CoA levels and alkaline mitochondrial pH promote non-enzymatic protein acetylation, independent of acetyltransferase enzymes [84].

Available evidence suggests a link between the metabolic state of the cell and histone acylation [85]. However, this relationship is highly affected by compartmentalization. It is not fully understood how the acetyl CoA is transported from mitochondria into the nucleus for histone acetylation modification as it cannot easily cross the mitochondrial membrane. Acetyl CoA export via citrate from the mitochondria and subsequent cleavage by ATP-citrate lyase in the cytosol is often suggested as the main source of acyl CoA in the nuclear-cytoplasmic compartment [79]. However, we have also recently shown an increased expression and activity of cytosolic carnitine acetyltransferase (CrAT) in hearts from HFD-fed mice [86]. This increase in CrAT activity can play a significant role in facilitating the transport and availability of acetyl CoA to the cytosol for acetylation and other cellular processes in the cytosol. Some researchers suggest acetyl CoA can be transported to the nucleus through nuclear pores from the cytosol [87]. However, this concept has not been adequately explored. A recent study by the Sutendra group revealed the presence of mitochondrial subunits of PDH in the nucleus and suggested that this PDH in the nucleus generates acetyl CoA essential for histone acetylation [88]. Interestingly, deletion of PDH decreases acetyl CoA synthesis from pyruvate in the nucleus and lowers histone acetylation, indicating the role of nuclear PDH in acetyl CoA production for histone acetylation [88]. However, it is not yet clear how pyruvate is transported into the nucleus for such processes.

The acetyl CoA contribution of each fuel substrate for histone acetylation is still poorly defined. Recently, McDonnell et al., using a stable isotope tracing in AML12 cells, demonstrated that fatty acid-derived acetyl CoA leads to a significant increase in histone acetylation, while high glucose levels (25 mM) only modestly increases histone acetylation, suggesting a dominant role of fatty acids in regulating histone acetylation [89]. Interestingly, the authors also found that these changes in histone acetylation were associated with the upregulation of some of the genes related to lipid metabolism [89]. However, it is worth mentioning that the authors used high levels of octanoate (2 mM), which is not a common fatty acid, unlike palmitate and oleate in the heart. Furthermore, these studies primarily used cancer cell lines, and the generalizability of the results to the heart needs further investigation.

## Regulation of protein lysine acetylation during obesity

### Lysine acetyltransferases

The acetylation process is regulated by two opposing enzymes: lysine acetyltransferases (KATs) and lysine deacetylases (such as HDACs) (Figure 2). KATs catalyze the transfer of acetyl groups from acetyl CoA onto the ε-amino groups of lysine residues of histone or non-histone proteins. KATs can be described as histone acetyltransferase (HATs) in the case of histone acetylation. Several HATs have been identified in relation to histone and other nuclear protein acetylation, which can be broadly classified as type A or type B based on their subcellular origin and function [90, 91]. Type A HATs are localized in the nucleus and involved in acetylation of histone and nuclear proteins, and are linked with the regulation chromatin conformation and gene transcription process. Type A HATs are further divided into MYST (MOZ, YBF2/SAS3, SAS2, and TIP60), GCN-5-related N-acetyltransferases (GNAT), and CREB-binding protein and p300 (CBP/p300) families, which contains several HAT sub-families [90].

Type B HATs are cytosolic HAT enzymes responsible for the acetylation of newly synthesized histones before they are transported and incorporated into the newly replicated DNA in the nucleus [90]. These HAT subgroups have diverse substrate specificities in histone or non-histone proteins. While each HAT has specific and different lysine residue targets on histones, there is a huge overlap in the protein substrate [92]. However, the specificity is also determined by other factors, including the sequence, structure and interactions with other coactivators or transcription factors [92]. These complex substrate specificity and functional redundancy in several cellular processes among HATs pose a significant challenge in developing effective therapies targeting HATs.

There are limited evidence regarding the roles of HATs in obesity and other metabolic diseases. However, recent studies revealed the important roles of MYST member, HAT8 or MOF, in maintaining metabolic homeostasis in adipose tissue in response to HFD [93]. It has been shown that MOF-mediated histone acetylation (H4K16ac) is a crucial regulator of *Pparg* and *Ppargc1a* gene expression, which are responsible for glucose uptake and fat storage in adipocytes [93]. Interestingly, the authors demonstrated that deletion of MOF showed resistance to fat mass gain in adipocytes after HFD. The study also indicated that MOF deletion is associated with a decreased glycolysis rate in the heart [93]. Other studies demonstrated a positive correlation between oleic-palmitic acid-induced lipid accumulation and HAT activity in HepG2 cells [94]. An increased HAT activity following fatty acid-induced lipid accumulation was associated with increased acetylation of histones (H3K9, H4K8, and H4K16) and non-histone proteins as well as in upregulation of lipogenic genes such as PPARγ, ACLY, and FASN [94]. These effects are effectively reversed by the addition of a p300/CBP-specific inhibitor, C-646^94^. Similarly, upregulation of P300/CBP has been shown in the liver after HFD feeding and in *ob/ob* mice [95]. P300/CBP, in turn, hyperacetylates insulin receptors (IRS1/2), which impairs insulin signalling [95]. On the other hand, P300/CBP inhibition with C646 improves insulin sensitivity and decreases hyperglycemia in obese mice [95].

There is limited data regarding KATs involvement in non-nuclear proteins. While it is widely suggested that mitochondrial protein acetylation may occur through non-enzymatic acetylation, some studies indicated that the GNAT family, general control of amino acid synthesis 5-like 1 (GCN5L1) acetyltransferase, may contribute to the mitochondrial protein acetylation changes [96, 97]. GCN5L1 activity depends on acetyl CoA mitochondrial production [98]. Its expression is upregulated in response to HFD in the liver and heart [99]. Hyperacetylation of mitochondrial proteins has been shown in association with increased GCN5L1 expression in HFD [99]. Specifically, GCN5L1 targets several fatty acid oxidation (FAO) enzymes and PDH in the mitochondria [99–101]. While GCN5L1-induced hyperacetylation promotes the activity of fatty acid oxidation enzymes [97, 99], it impairs PDH [100]. On the contrary, GCN5L1 deletion in cardiomyocytes decreases mitochondrial acetylation levels [97, 99] and fatty acid oxidation while improving glucose oxidation in HFD [100]. We have also shown that GCN5L1 is vital in the maturation of mitochondrial fatty acid metabolism in newborn hearts [102]. Its expression increases during the newborn period, resulting in hyperacetylation of key fatty acid oxidation enzymes, LCAD and β-HAD. This leads to increased rates of fatty acid β-oxidation and the maturation of mitochondrial cardiac energy metabolism [102].

On the contrary, it is believed that the cytosolic protein acetylation requires the involvement of KATs. However, the specific KATs regulating cytosolic protein acetylation (acylation) modification remain unclear. Some studies suggest that HATs, such as p300/CBP families, may shuttle between the nucleus and cytoplasm and regulate the cytoplasmic acetylation processes [63, 103, 104]. Other studies indicated that type B HAT, KAT1, α-tubulin N-acetyltransferase 1 (ATAT1) and N-terminal acetyltransferases 10 and 60 (NAA10 and NAA60) are also cytoplasmic KAT enzymes [90, 105].

### Histone deacetylases (HDACs)

Deacetylation is catalyzed by a group of HDACs. HDACs remove acetyl groups from ε-amino groups of lysine residues histone and non-histone protein substrates. Eighteen different HDACs have been characterized and grouped into 4 major classes based on sequence similarities: class I (HDACs 1-3, 8), class II (HDACs 4-7, 9, 10), class III (sirtuins or SIRTs 1-7), and class IV (HDAC11) (Figure 2) [90]. HDACs class I, II, and IV are described as classical HDACs and are dependent on zinc as a co-factor for their deacetylase activity. Classical HDACs regulate key aspects of cellular processes, including metabolism, inflammation, and vascular function, through altering chromatin structure and gene expression by deacetylation of histone proteins [106]. In addition, HDACs can also control the deacetylation of non-histone proteins in or outside of the nucleus. In support of this, HDAC1 and HDAC2 have been detected in the mitochondrial isolates from mouse hearts [107]. Class III HDACs are NAD^+^-dependent deacetylases, also known as sirtuins or SIRTs, that can act as deacetylases outside the nucleus.

Emerging evidence on the relationship between obesity and HDACs highlights the complex and bidirectional mechanisms. Obesity influences HDAC expression levels and activities, leading to dysregulations in energy metabolic pathways, insulin sensitivity and adipogenesis. For instance, studies by Tian *et al.* [108] and Bricambert *et al.* [109] demonstrated that obesity, induced by both dietary and genetic interventions, upregulates HDAC8 activity in the liver during NAFLD, and HDAC5/6 in adipocytes, respectively. Increased activity of HDAC8 has also been associated with insulin resistance [108], while high HDAC5/6 activities led to adipocyte dysfunction [89].

Improvements in metabolic parameters have been shown in different obese models in response to HDAC inhibition. For instance, HDAC11 deletion in mice prevented obesity after HFD feeding, and significantly improves insulin sensitivity and glucose tolerance [110]. Some of these protective effects were attributed to increased expression and activity of UCP protein in adipose tissue [110]. Additionally, sodium butyrate (a pan HDAC inhibitor) treatment in obese mice led to an improved insulin sensitivity, adiposity reduction and increased energy expenditure [88]. Similar results were also observed with class I HDAC inhibition with MS-27 in HFD-fed obese mice [73]. Likewise, other studies have demonstrated improved glycemia and insulin secretion in obese diabetic rats in response to HDAC3 inhibition [72].

One of the hallmarks of obesity is an increase in leptin-resistant adiposity, which consequently leads to adipocyte dysfunction [111]. Interestingly, HDAC6 inhibition in *db/db* and obese mice increases leptin sensitivity and decreases obesity [112]. Furthermore, a negative association between HDAC1 activity and brown adipocyte thermogenesis has been shown, which was linked to histone acetylation changes and gene expression patterns [113]. Altogether, these findings offer insights into the therapeutic potential of targeting different HDACs to treat obesity. However, most of these studies were performed in non-cardiac tissues, mainly liver and adipose tissue. Although this has an indirect implication for the heart, the impact of obesity on cardiac HDAC expression/activity and the role of these alterations on obesity-induced cardiac metabolic perturbations and cardiac dysfunction remains to be elucidated.

### Sirtuins (SIRTs)

Sirtuins are responsible for the deacetylation of cytoplasmic and mitochondrial proteins [114, 115]. Seven mammalian sirtuin proteins (SIRT1-SIRT7) have been identified [116]. SIRT2 predominantly functions in the cytoplasm [117], while SIRT1, 6, and 7 reside mainly in the nucleus [116, 118, 119]. SIRT 3, 4 and 5 are major mitochondrial deacetylases (Figure 2) [116, 120, 121]. However, this compartmentalization is not exclusive, and each sirtuin may shuttle across cellular compartments and regulate the acetylation state of diverse cellular proteins [118, 122–124]. While SIRT 1-3 possesses potent deacetylase activity [125–127], SIRT 4-7 have weak or no detectable deacetylase activity or have a high specificity for selective acetylation substrates [125, 128]. SIRT5 has potent lysine demalonylation and desuccinylation activity [124, 129, 130]. Altogether, sirtuins regulate diverse processes, including metabolism, gene expression, cell survival and several other processes in various tissues [131].

Obesity is associated with the reduction in expression or activity of some sirtuins. For instance, we have previously shown a decreased cardiac SIRT3 expression in HFD-fed mice, which was associated with cardiac protein hyperacetylation [35]. Similarly, significantly reduced SIRT3 expression has also been found in patients with obesity and HF [72]. The authors demonstrated a negative correlation between protein hyperacetylation and reduced SIRT3 expression [72]. In the hearts of SIRT3 KO mice fed HFD, we have shown a significant increase in cardiac protein acetylation, including the hyperacetylation of LCAD and β-HAD [35]. This acetylation change is accompanied by a shift in cardiac energy metabolism toward high rates of fatty acid oxidation [35]. Similarly, HFD-feeding in SIRT3 KO mice led to hyperacetylation of the PDH enzyme and suppression of glucose oxidation in skeletal muscle [84].

A reduction in SIRT1 expression and activity associated with HFD-induced obesity has also been reported in various studies [100, 108]. Intriguingly, some studies suggest that weight loss through calorie restriction correlates with increased SIRT1 expression [100]. However, there are also conflicting results regarding the effects of SIRT1 in obesity-induced metabolic dysfunction. For instance, Xu et al. [101] found brown adipose tissue (BAT) degeneration and exacerbated dysfunction in response to SIRT1 deficiency in mice, while studies by White et al. [132] reported no beneficial effects of SIRT1 overexpression on HFD-induced glucose intolerance, weight gain, or insulin resistance. Other studies have shown that SIRT6 expression is reduced in adipose tissue both in HFD-fed and *ob/ob* mice as well as in aged mice [133]. On the contrary, adipose tissue-specific deletion of SIRT6 sensitized mice to HFD obesity and led to a decreased adipose triglyceride lipase (ATGL) levels due to acetylation changes on its transcriptional regulator, FOXO1 in the SIRT6 KO mice [133].

NAD^+^ is a critical co-substrate for the sirtuins. Some studies have demonstrated an association between HFD or obesity and decreased NAD^+^ levels in the heart [113]. On the contrary, other studies indicated that increasing NAD^+^ levels through NR supplementation enhances SIRT1 and SIRT3 activity and protects against HFD-induced metabolic abnormalities [134].

## Contribution of protein acetylation to cardiac metabolic alterations in obesity

One of the most notable findings in the mass discovery of non-histone protein acetylation is the abundance of acetylation modifications on energy metabolic enzymes (Figure 3). Since the first landmark study by Kim et al. in 2006 [135], numerous acetylated proteins have been identified in the cytosol and mitochondria [118, 119]. Of these acetylated proteins, fatty acid and glucose metabolic enzymes are abundantly represented [127]. For instance, all of the enzymes involved in fatty acid β-oxidation, including long-chain acyl CoA dehydrogenase (LCAD), enoyl-CoA hydratase, L-3-hydroxy acyl-CoA dehydrogenase (β-HAD), and 3-ketoacyl-CoA thiolase (3-KAT), are subjected to acetylation modification (Figure 3) [35, 75]. In addition, other proteins involved in fatty acid transport and metabolism have been identified with acylation modification [128]. Similarly, acetylation of key enzymes of glucose oxidation, such as PDH, has been reported [84, 100]. Moreover, at every step of glycolysis, glycolytic enzymes are subjected to acetylation modifications [136]. Although acetylation modifications are widespread among metabolic enzymes, its real impact on enzyme activity and metabolic flux in glucose and fatty acid metabolism are still incompletely understood. In the following section, we will discuss recent findings on the impact of protein acetylation on cardiac energy metabolism in association with obesity.

## Impact of acetylation on myocardial fatty acid oxidation rates

Increased acetylation of myocardial fatty acid oxidation enzymes, including LCAD and β-HAD, in response to a HFD, obesity and diabetes have been reported [35, 101, 137, 138]. Similarly, increased acetylation of fatty acid oxidation enzymes has been observed in HFpEF hearts [139] and in the liver of obese animals [74, 140, 141]. However, there are conflicting views regarding the actual impact of acetylation on the fatty acid oxidation enzyme activities and fatty acid oxidation rates in the heart. In HFD-fed and SIRT3 KO mice, we have shown that chronic HFD led to an overall myocardial protein hyperacetylation as well as increased acetylation of myocardial LCAD and β-HAD [35]. Importantly, increased acetylation of these enzymes was positively correlated with their activity and increased myocardial fatty acid oxidation rates (Table 2) [35]. In a separate study, we have also replicated the same results in obese mice subjected to transverse aortic constriction (TAC) induced HF [38]. Interestingly, weight loss in these obese mice decreased the acetylation of these enzymes and fatty acid oxidation rates [38].

**TABLE 2:** Effects of lysine acetylation on cardiac fatty acid and glucose metabolic enzymes.

Metabolic pathways	Target enzymes	Effects on enzyme activity	References
Fatty acid oxidation	LCAD	Increased, Decreased	[35, 97, 139, 142, 143]
	β -HAD	Increased, Decreased	[35, 97, 139, 143]
	MCAD	Increased	[144]
	MCD	Increased	[128]
Glucose oxidation	PDH	Decreased	[35, 38, 97, 100, 143]
	MPC	Decreased	[83]

GLUT4, glucose transporter isoform 4; MCT, monocarboxylate transporter 1; PDH, pyruvate dehydrogenase; LCAD, long-chain acyl CoA dehydrogenase; β–HAD, β-hydroxyacyl CoA dehydrogenase; MCAD, medium-chain acyl CoA-dehydrogenase; MCD, malonyl CoA decarboxylase; MPC, mitochondrial pyruvate carrier; PGM, phosphoglucomutase; HK, hexokinase.

Other studies have also shown a correlation between hyperacetylation and increased activities of cardiac fatty acid oxidation enzymes (Table 2) [101]. In mice fed a HFD for 24 weeks, Thapa et al. found an increased expression of GCN5L1 along with hyperacetylation of fatty acid oxidation enzymes [101]. The authors further demonstrated that deletion of GCN5L1 in H9c2 decreased the acetylation status and activity of fatty acid oxidation enzymes [101]. We have also shown a decrease in fatty acid oxidation and acetylation of fatty acid oxidation enzymes in newborn hearts that have undergone hypertrophic remodelling [145]. Furthermore, deletion of the GCN5L1 in H9c2 cells or hypertrophic remodelling of newborn hearts leads to a decreased myocardial acetylation and impaired maturation of myocardial fatty acid oxidation [145].

A positive association between acetylation and fatty metabolism has also been shown in the hearts of diabetic animals [83, 144, 146–148]. In both type 1 and type 2 diabetes, increased acetylation of myocardial fatty acid metabolic enzymes promotes their enzyme activity and is associated with cardiac metabolic inflexibility during diabetes [147, 149]. Further evidence supporting the link between hyperacetylation and enhanced activity of fatty acid metabolic enzymes has been found in the liver and skeletal muscles. For instance, the association between excessive acetylation and increased palmitate oxidation rates has been observed in skeletal muscle in mice lacking SIRT3 [84]. Similar results have also been reported in liver cells exposed to high fat and deacetylase inhibitor [68]. Altogether, these data suggest that hyperacetylation of cardiac fatty acid oxidation enzymes in obesity and diabetes has a stimulatory effect on fatty acid oxidation. Thus, acetylation may contribute to cardiac metabolic inflexibility characterized by increased heart’s reliance on fatty acid oxidation during obesity.

On the contrary, an inhibitory effect of hyperacetylation on fatty acid metabolism has also been suggested in various studies [69, 139, 142, 143, 150]. In mice with HFpEF, induced by a two-hit model of a chronic HFD and hypertensive stress (L-NAME treatment), Tong et al. showed an increased acetylation of cardiac fatty acid oxidation enzymes [139]. By measuring the activities of some of the fatty acid oxidation enzymes in isolated mitochondria, the authors suggested that hyperacetylation is associated with reduced activity of fatty acid oxidation enzymes and impaired mitochondrial fatty acid metabolism [139]. However, this contrasts with recent evidence showing an actual increase in myocardial fatty acid oxidation rates in HFpEF hearts [15, 151]. Similarly, reduced activity of LCAD due to hyperacetylation has been shown in the liver of SIRT3 deficient mice [69]. In the same study, the authors also showed that increasing SIRT3 expression through fasting decreased LCAD acetylation and increased its enzyme activity [69].

Various factors may contribute to the conflicting results regarding the effects of acetylation on fatty acid oxidation enzymes. Firstly, the effect of acetylation and its regulation is complex and context-dependent. Thus, the impact of acetylation can vary depending on the specific disease conditions, target cells/organs, and substrate availability or metabolic state. Secondly, the methodological variation used to measure fatty acid oxidation or enzyme activity may also contribute to the discrepancies in these studies. For instance, some studies used isolated muscle fibers/mitochondria to measure the effect of acetylation on enzyme activity. However, these cells are in a quiescence state, and the important role of workload on fatty acid oxidation is missing. The necessary signalling pathways are also lacking, which could affect the overall outcome of the study. Additionally, the composition and substrate concentration of buffers used to determine metabolic rates could affect the accuracy of such measurements. Some studies used non-physiological concentrations involving single substrates or imbalanced free fatty acid to albumin ratio, which can influence the rate of fatty acid metabolism.

## Impact of acetylation on cardiac glucose oxidation rates

Unlike fatty acid oxidation, most researchers agree that acetylation has an inhibitory effect on glucose oxidation (Table 2). Increased acetylation of the cardiac PDH enzyme occurs in obesity [38, 100, 101]. A recent study showed that a long-term HFD in aged mice led to a diastolic dysfunction, a pre-HFpEF state, and an increased acetylation of cardiac PDH [100]. This hyperacetylation of PDH was inversely correlated with its enzyme activity [100]. Furthermore, the acetylase GCN5L1 was also shown as a regulator of PDH acetylation and activity, as its deletion reversed the hyperacetylation of PDH and increased PDH’s enzyme activity [100]. Similarly, previous studies from our lab have also shown obesity-induced hyperacetylation of PDH in the heart, which is associated with a marked decrease in cardiac glucose oxidation rates [38]. In contrast, switching to a low-fat diet or caloric restriction significantly reduces PDH acetylation status and enhances glucose oxidation rates in the heart [38]. In addition to the heart, other studies have also revealed a negative impact of acetylation on PDH in skeletal muscles in mice lacking SIRT3 [84]. SIRT3 deletion impairs glucose oxidation in skeletal muscle and leads to lactate accumulation and a shift towards excessive fatty acid oxidation [84]. Importantly, it was also shown that increased acetylation of PDH promotes its phosphorylation, which further suppresses its enzyme activity.

In addition to PDH, increased acetylation of the mitochondrial pyruvate carrier protein (MPC) has also been shown in diabetic mice hearts. It was shown that hyperacetylation of MPC leads to a significant decrease in its function, as indicated by impaired pyruvate uptake and suppressed pyruvate-based mitochondrial respiration [83]. Overall, these studies suggest the potential contribution of acetylation to impaired cardiac glucose oxidation in HFD and obesity. Together with the altered acetylation of fatty acid oxidation enzymes, which has a stimulatory effect on fatty acid oxidation, the acetylation dysregulation in HFD and obesity may potentially contribute to cardiac energy metabolic perturbations seen in obese subjects. Hyperacetylation of cardiac metabolic enzymes during obesity may lead to a metabolic rewiring characterized by increased fatty acid oxidation and decreased glucose oxidation.

## Acetylation as a therapeutic approach in obesity-induced cardiac metabolic alterations

### HDACs as a therapeutic target in obesity

In recent years, HDAC inhibitors have gained significant research attraction as a potential therapeutic option for various diseases. Numerous HDAC inhibitors have been developed and several others are being investigated for various therapeutic applications, including for HF and metabolic diseases [152]. HDAC inhibitors have also been proven to be effective and promising for certain cancer treatments. Vorinostat (class I and II HDAC inhibitor) was the first HDAC inhibitor to gain FDA approval in 2006 for the treatment of cutaneous T-cell lymphoma (CTCL). Since then, three other HDAC inhibitors, panobinostat (pan-HDAC inhibitor), belinostat (pan-HDAC inhibitor) and romidepsin (class I HDAC inhibitor), have also been approved by FDA for the treatment of hematological malignancies [137].

Emerging evidence suggests that HDAC inhibitors have therapeutic potential for a wide variety of diseases, including HF and obesity. Several studies indicated the promising therapeutic effects of HDAC inhibitors against cardiac hypertrophy [138, 153] and myocardial ischemia and reperfusion injury [140, 141, 154]. As discussed, changes in KAT and HDAC may contribute to the pathogenesis of obesity-induced metabolic alterations during HF. Furthermore, these studies have also shown that HDAC can affect the expression of genes involved in adipogenesis, lipolysis and energy metabolism. In fact, several animal models of obesity and HFD have also demonstrated that certain HDAC inhibitors can effectively reduce adiposity, improve leptin sensitivity, increase energy expenditure, and improve insulin sensitivity and glucose homeostasis in obese animals. This suggests that HDAC inhibitors are a potentially promising avenue for the treatment of obesity and related metabolic alterations. However, similar studies are lacking in obesity-related HF.

There are still several challenges in improving the specificity of HDAC inhibitors. HDACs have complex substrate specificity, functional redundancy and multidimensional interaction with other proteins that can affect their specific action. Often, multiple transcriptional and non-transcriptional mechanisms are involved in HDAC inhibitors’ mechanism of action. For instance, several pathways, including cell proliferation, differentiation, inflammation and apoptosis across different tissues and organs, may be affected by HDAC inhibitors, which may lead to potential off-target effects. Additionally, several HDAC isoforms are shown to affect the pathogenesis of obesity. Thus, for better outcomes, it is crucial to elucidate the most significant underlying pathogenesis mechanisms modified by HDAC inhibitors in relation to obesity-induced cardiac metabolic alterations. Further research is needed to identify a subset of HDACs that are more relevant for treating obesity and metabolic alterations in HF.

### Sirtuins as a therapeutic target in obesity

The discovery that protein acetylation is a widespread PTM modification of metabolic proteins and is dysregulated in various diseases has attracted interest in the development of acetylation and/or sirtuin modulators as therapeutic tool. Several plant extracts, such as honokiol, resveratrol, quercetin, curcumin, and berberine have been developed [146]. Similarly, numerous synthetic small molecules such as SIRT1-activating compounds (STACs), SRT1720, and SRT2104 have been developed to modulate sirtuin activities [145, 146]. Despite the availability of many of these sirtuin activators and inhibitors, most of them suffer from low specificity, lack of a unique target, weak potency, and unclear mechanism of action. There is still a lack of comprehensive data and consensus on the effective pharmacological activator of sirtuins. This poses a significant challenge in studying the translational potential of targeting sirtuins/acetylation modulation as a therapeutic option for the treatment of cardiac metabolic alterations.

### Acetylase inhibition as a therapeutic target in obesity

Comparatively, there are few available KAT inhibitors. As KATs often form large complexes with other proteins, targeting them selectively is challenging [155]. In addition, the structural and functional diversity of KATs complicates the development of specific drugs targeting each KAT^90^. There are naturally occurring pan-HAT inhibitors derived from plants, such as anacardic acid, garcinol, and curcumin [155]. While anacardic acid and garcinol inhibit P300/CBP and PCAF HAT enzyme activities [156], and curcumin selectively inhibits P300/CBP [155]. However, there is a lack of well-designed pre-clinical animal studies on the effects of these HAT inhibitors in disease settings such as obesity. Besides, these HAT inhibitors are poorly soluble and have poor cell permeability, and their pharmacokinetics are not fully characterized [155]. Recently, new small molecule or synthetic HAT inhibitors, including C646 and WM-1119, have been developed [157]. While these new promising drugs offer more specificity and potency, further research is needed to evaluate the effectiveness of these new generations of HAT inhibitors in specific disease models.

## Discussion

Obesity is associated with hyperacetylation of several cardiac energy metabolic enzymes, including those involved in fatty acid oxidation and glucose oxidation. Hyperacetylation during obesity may contribute to cardiac metabolic inflexibility by stimulating fatty acid oxidation and suppressing glucose oxidation. Although acetylation modulation holds a potential therapeutic value, there is still a lack of well-designed studies with rigorous experimental approaches and adequately validated acetylation-modulating drugs in relevant disease models.
